# Antimicrobial Resistance and Virulence Gene Profiles of Methicillin-Resistant and -Susceptible *Staphylococcus aureus* From Food Products in Denmark

**DOI:** 10.3389/fmicb.2019.02681

**Published:** 2019-12-13

**Authors:** Heng Li, Paal Skytt Andersen, Marc Stegger, Raphael N. Sieber, Hanne Ingmer, Nicholas Staubrand, Anders Dalsgaard, Jørgen J. Leisner

**Affiliations:** ^1^Department of Veterinary and Animal Sciences, Faculty of Health and Medical Sciences, University of Copenhagen, Copenhagen, Denmark; ^2^Department of Bacteria, Parasites and Fungi, Statens Serum Institut, Copenhagen, Denmark; ^3^School of Chemical and Biomedical Engineering, Nanyang Technological University, Singapore, Singapore

**Keywords:** MRSA, MSSA, retail meat, ready-to-eat food, antibiotic resistance, toxin genes, CC types, *Staphylococcus aureus*

## Abstract

Foods may potentially serve as vehicles for the transmission of antimicrobial-resistant variants of *Staphylococcus aureus* that are important in a human clinical context. Further, retail food products can be a cause of staphylococcal food poisoning. For these reasons and to account for source attribution and risk assessment, detailed information on the population structure, resistance, and virulence profiles of *S. aureus* originating from retail food products is necessary. In the current study, whole-genome sequences from 88 *S. aureus* isolates were subjected to bioinformatics analyses in relation to sequence types, antimicrobial resistance, and virulence profiles. The sequence types (ST) identified belonged to 13 clonal complexes (CC) with CC5 and CC398 being the most common. CC398 was identified as the dominant clone (*n* = 31). CC5 was identified as of avian origin, with the presence of φAVβ prophage genes (*n* = 13). In total, 39.8% of the isolates contained multiple resistance genes, and methicillin-resistant *Staphylococcus aureus* (MRSA) isolates were found in CC8, CC9, and CC398. Genes conferring resistance to the antimicrobial classes of β-lactams, tetracycline, and erythromycin were detected in this study, all of which are commonly used in Danish livestock production. The *tst* gene encoding the toxic shock syndrome toxin was for the first time identified in ST398 isolates, probably as a result of a single acquisition of a SaPI-like element. The sushi-CC398 isolates carrying the *scn* gene likely originated from a human reservoir, while the other isolates originated from livestock. Taken together, our results show that both human and animal reservoirs contribute to contamination in food products and that retail foods may serve as a vehicle of *S. aureus* between livestock and humans.

## Introduction

*Staphylococcus aureus* is an opportunistic pathogen causing severe conditions such as endocarditis, toxic shock syndrome, scalded skin syndrome, and osteomyelitis (Otto, 2014). It is found in raw food of animal origin but also in, e.g., ready-to-eat foods as a result of contamination during the handling process and may cause food poisoning if present in high numbers (Le et al., 2003). The prevalence of antimicrobial-resistant and especially methicillin-resistant variants (MRSA) is a major human health concern, in particular in hospitals, and MRSA is increasingly found in food products. Also, livestock-associated MRSA (LA-MRSA) has been isolated from animal-derived foods such as turkey and pork meat (Mossong et al., 2015; Tang et al., 2017). MRSA variants exhibit resistance to β-lactams through the acquisition of the mobile staphylococcal cassette chromosome *mec* (SCC*mec*) (Ito and Hiramatsu, 1998). Methicillin-susceptible *S. aureus* (MSSA) of human or animal origin is found in a broad range of foods and is frequently resistant to a range of antimicrobials such as tetracycline, erythromycin, and gentamicin (Waters et al., 2011).

*Staphylococcus aureus* harbors genes encoding a variety of virulence factors including enterotoxins (SEs; *sea* to *see*, *seg* to *sei*, *ser* to *set*), exfoliative toxins (*eta*, *etb*), toxic shock syndrome toxin-1 (*tst*), Panton-Valentine leukocidin (*lukS/F*-*PV*), staphylococcal complement inhibitor (*scn*), and hemolysins (*hly/hla*, *hlb*, *hld*, *hlgA*, *hlgB*, *hlgC*) (Chen et al., 2005; Argudín et al., 2010)^1^. Enterotoxin production is important as it causes food poisoning, whereas the other virulence factors are associated with infection rather than intoxication. The *scn* gene is a marker of the immune evasion cluster (IEC) of strains that has been detected at high frequency among diverse collections of *S. aureus* strains obtained from humans, indicating that the *scn* gene may be useful for differentiating strains that have been transmitted from livestock to humans from those of human origin (Rinsky et al., 2013).

In Denmark, the surveillance of MRSA is established by the Staphylococcus group at Statens Serum Institut^2^ and recorded by the DANMAP initiative^3^, with results published on a yearly basis. All isolates are tested by multiplex PCR for the presence of genes encoding staphylococcal protein A (*spa*), *mecA*, CC398 (*sau1*-*hsd*), staphylococcal complement inhibitor (*scn*), and *lukF*-PV (DANMAP, 2017). Except for the *lukF*-*PV* encoding gene, isolates are not routinely examined for the presence of other virulence genes such as enterotoxins and exfoliative toxins (Franck et al., 2017).

With the aim of determining the livestock or human origin of MRSA and MSSA isolated from foods sold in Denmark, a bioinformatics approach was employed to study the relatedness of *S. aureus* isolated from animals (chicken, pork, and turkey processed retail meat) or from foods subjected to human handling (sushi and pasta salads). Further, we examined genotypic antimicrobial resistance profiles and possession of virulence factors in order to conduct an overall evaluation of the potential risks associated with the presence of MRSA and MSSA in foods.

## Materials and Methods

### *Staphylococcus aureus* Strains

A total of 88 *S. aureus* isolates were included in this study, including 29 isolates from 17 types of raw chicken products, 15 from six types of raw turkey products, and 17 from 10 types of raw pork products. The products were obtained randomly from Danish supermarkets located in the area of Copenhagen between November 2014 and September 2015 (Tang et al., 2017). The meat was of Danish origin except for the turkey, which was presumably imported from Germany. In addition, 17 strains were selected representing the different *spa*-types observed among isolates obtained from sushi, i.e., Maki and Nigiri with salmon, from ready-to-eat restaurants representative of positive or negative food inspector rankings in Copenhagen during December 2017 and January 2018 (Li et al., 2019). Finally, ten isolates were obtained in January and February 2018 from pasta salads from a Danish outlet using the same sampling and isolation methods as employed for the sushi isolates. This outlet was selected due to a previous study showing high bacterial counts for the pasta salad products (Kjeldgaard et al., 2010; the shop was listed as outlet “C”). Among the 88 isolates, 14 isolates were identified as MRSA, as previously reported (Tang et al., 2017).

### Whole-Genome Sequencing

Isolates were grown in Luria-Bertani broth (240230; Difco, United States) for 24 h at 37°C while being shaken, and genomic DNA was extracted and purified using a DNeasy Blood & Tissue Kit, according to the manufacturer’s instructions (Qiagen GmbH, Hilden, Germany). The quality of the extracted DNA was assessed using a Nanodrop ND-1000 spectrophotometer (Nanodrop Technologies, Wilmington, DE, United States) and 1.0% (w/v) agarose gel. Purified DNA was whole-genome sequenced using Illumina’s NextSeq 500 (Illumina, San Diego, CA, United States). Genome assemblies were obtained using SPAdes v3.9 (Nurk et al., 2013), and the quality was evaluated with QUAST v2.3 (Gurevich et al., 2013). The raw sequencing reads from the strains are available at the European Nucleotide Archive under project accession ID PRJEB32298.

### Analysis of MLST and Clone Complexes

Multilocus sequence typing was obtained using the MLST 2.0 tool (Larsen et al., 2012) on the Illumina read files and eBURST v3^4^ analysis was performed to group STs into clonal complexes (CCs). A Minimum Spanning Tree (MST) based on ST types was created using BioNumerics v7.5 (Applied Maths, Sint-Martens-Latem, Belgium). A single nucleotide polymorphism (SNP) tree for all isolates was constructed by CSI Phylogeny v1.4, available at the Center for Genomic Epidemiology^5^, and visualized with genotypic data using PhyD3 (Kreft et al., 2017).

### Construction and Analysis of Phylogenetic Tree for CC398 Isolates

A phylogenetic tree was constructed using all CC398 isolates obtained from the Danish food products in this study (*n* = 14) and the CC398 isolates from a previous study on LA-MRSA CC398 in Denmark (Sieber et al., 2018) including isolates from Danish pig farms (*n* = 209), Danish patients (*n* = 83), people who were registered in the national MRSA database as having had occupational contact with livestock and having been colonized or infected with LA-MRSA CC398 (*n* = 79), and isolates from the international reference collection (*n* = 82) (Price et al., 2012). SNPs were identified by mapping reads against the ST398 reference genome (strain S0385; GenBank accession no. AM990992) through Northern Arizona SNP Pipeline (NASP) (Sahl et al., 2016). SNPs falling into regions of putative recombination, such as the ∼123-kb region that was horizontally acquired from a CC9 donor (Price et al., 2012), were removed from the alignment. The maximum-likelihood phylogenetic tree was established in IQTREE (Nguyen et al., 2014) from the remaining sites using default settings. The robustness of the phylogeny was assessed with the software’s Ultrafast bootstrap method using 1,000 replicates. Finally, the phylogenetic tree was imported to iTOL (Interactive tree of life) for visualization (Letunic and Bork, 2016)^6^.

### Identification of Antimicrobial Resistance and Virulence Genes

Antimicrobial-resistant genes (Zankari et al., 2012) and virulence genes (Joensen et al., 2014) were identified with the online tools ResFinder and VirulenceFinder^7^. For a hit to be reported by the two programs, it had to cover at least 60% of the length of the gene sequence in the database with a sequence identity of 60 and 90%, respectively. The SAAV_2008 and SAAV_2009 genes associated with the avian φAVβ prophage were extracted from the NCBI database (GenBank id NC_013450) and searched for in the new isolates of this study with BLASTN in CLCbios’s Genomics Workbench v6.5. The criterion for determining gene presence was set at ≥95% identity between the query gene and the reference sequence.

## Results and Discussion

### Sequence Types and Clonal Complex of *S. aureus* Isolates From Food Products

The MLST typing of the 88 *S. aureus* genomes revealed a total of 17 distinct ST types, with the most commonly detected being ST398 (35%) and ST5 (15%). MSSA isolates (*n* = 74) were detected among all ST types, while MRSA isolates (*n* = 14) were only found among ST8, ST9, and ST398 from retail meat. Thirteen CC types were identified according to eBURST V3, namely CC1, CC5, CC7, CC8, CC9, CC15, CC20, CC30, CC45, CC88, CC101, CC398, CC779, and one ST2867 (see Table 1 for CCs and STs).

**TABLE 1:** CC, MSSA/MRSA, ST, and *spa* profiles of *S. aureus* isolates from retail meat and ready-to-eat foods.

**CC type**	**MSSA/MRSA**	**ST^a^**	***Spa* type^b^**	**Source^c^**
CC1	4/0	ST1 (4)	t127 (1), t273 (2), ND (1)	Chicken (2), pork (1), sushi (1)
CC5	13/0	ST5 (13)	t034 (1), t3478 (11), ND (1)	Chicken (13)
CC7	6/0	ST7 (6)	t605 (2), t091 (2), t11399 (2)	Pork (2), sushi (4)
CC8	0/3	ST8 (3)	t008 (3)	Chicken (3)
CC9	4/2	ST9 (5)	t337 (1), t1430 (2), t6158 (2)	Pork (3), turkey (2)
		ST2423 (1)	t15045 (1)	Pork (1)
CC15	3/0	ST15 (3)	t084 (3)	Chicken (1), pasta salad (2)
CC20	2/0	ST1281 (2)	t458 (1), ND (1)	Sushi (2)
CC30	7/0	ST30 (1)	t021 (1)	Pasta salad (1)
		ST433 (6)	t1273 (1), t1333 (5)	Chicken (5), pork (1)
CC45	7/0	ST45 (5)	t132 (1), t282 (1), t362 (1), t728 (1), ND (1)	Pork (1), sushi (4)
		ST972 (2)	t230 (2)	Pasta salad (2)
CC88	1/0	ST88 (1)	ND (1)	Sushi (1)
CC101	3/0	ST101 (3)	t056 (3)	Pasta salad (3)
CC398	22/9	ST398 (31)	t011 (2), t034 (18), t108 (1), t1451 (2), t2582 (1), t3478 (2), t3625 (1), ND (4)	Chicken (9), pork (4), turkey (13), sushi (4), pasta salad (1)
CC779	1/0	ST779 (1)	t878 (1)	Pasta salad (1)
Not determined	1/0	ST2867 (1)	t9602 (1)	Sushi (1)

*^*a*^Numbers in parentheses are the number of isolates per sequence (ST) type. ^*b*^Numbers in parentheses are the number of isolates per *spa* type. ND: not done (not typeable). ^*c*^Numbers in parentheses are the number of isolates from each source of food products.*

**FIGURE 1:**
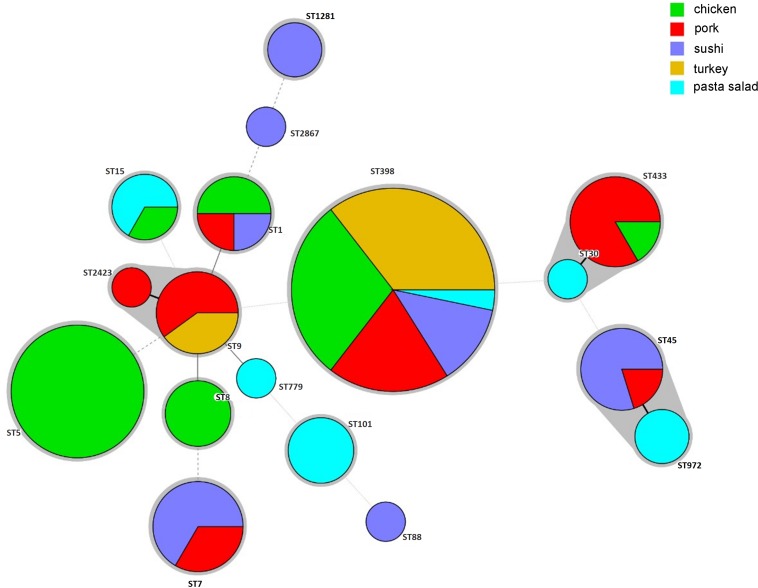
Minimum-spanning tree, as constructed from the MLST data of 88 *S. aureus* isolates collected from retail food products in Denmark. Circle sizes represent the number of isolates, and circle areas are colored by food source and labeled with sequence types (STs). The different colors represented isolates from retail chicken (*n* = 29), pork (*n* = 19), sushi (*n* = 17), turkey (*n* = 13), and pasta salad (*n* = 10).

The sources of different ST isolates in the CC groups are shown in Figure 1. ST398 was most common and originated from turkey (*n* = 13), chicken (*n* = 9), pork (*n* = 4), sushi (*n* = 4), and pasta salad (*n* = 1). ST5 and ST8 isolates were isolated from chicken products only. ST20 and ST88 were isolated from sushi. ST101 and ST779 were isolated from pasta salad. ST1, ST30, and ST45 were found in either chicken, pork, or sushi products. ST7, ST9, and ST15 were isolated from one or more of the sources chicken, pork, turkey, sushi, and pasta salad.

**FIGURE 2:**
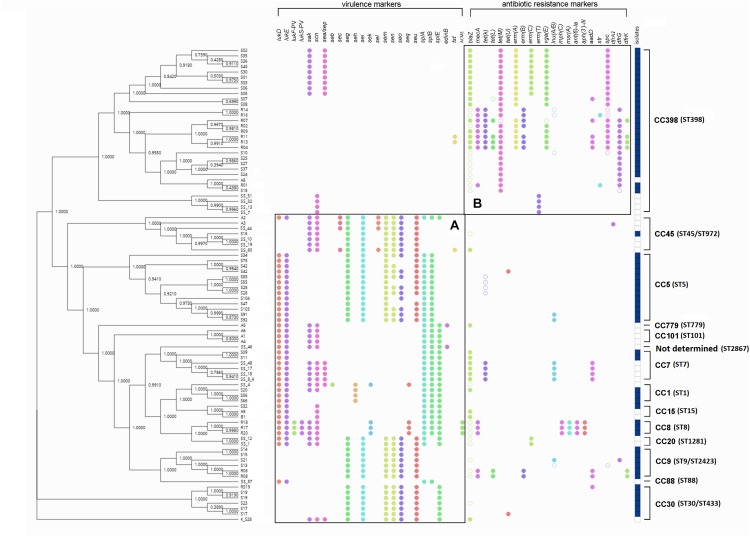
SNP cladogram of all *S. aureus* study isolates as obtained by the PhyD3 JavaScript library. The presence of virulence and antimicrobial resistance markers, isolate sources, as well as clonal complexes, is indicated on the right. The filled circles indicate confirmed markers with 100% identity and empty circles indicate potential markers with a 90% identity threshold, as extracted from ResFinder. The filled dark blue squares in the column to the right indicate a livestock origin, while the empty squares indicate a human-handling origin. **(A)** Virulence markers were located in various CC groups. **(B)** Antimicrobial resistance markers were mainly located on CC398, especially the livestock-associated CC398 with the dark blue square markers.

Associations were observed for different CC types/STs and the presence of genes encoding virulence or antimicrobial resistance. Thus, virulence genes were present in various clonal complex groups (Figure 2A), while antimicrobial resistance genes were present mostly in CC398 isolates, especially in livestock-associated CC398 isolates (Figure 2B).

**FIGURE 3:**
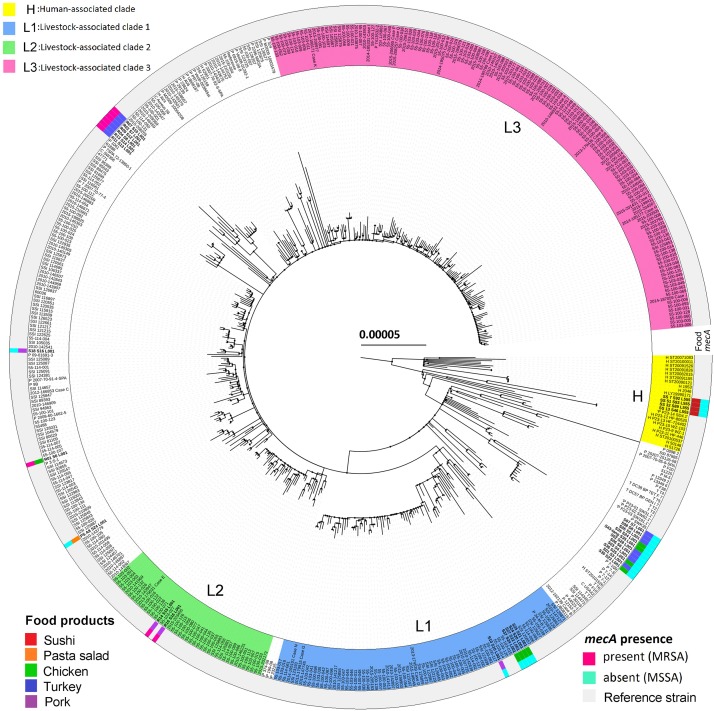
Maximum-likelihood phylogeny as obtained from the 31 *S. aureus* CC398 isolates from this study and 453 *S. aureus* CC398 isolates from Sieber et al. (2018). The tree was rooted according to the work of Price et al. (2012). The scale bar represents the number of nucleotide substitutions per variable site. The type of food products and methicillin-resistance gene *mecA* are shown. The basal human-associated lineage of *S. aureus* CC398 (H), according to Price et al. (2012), as well as the three most prevalent lineages in Danish pigs (L1, L2, and L3), according to Sieber et al. (2018), are highlighted.

In Denmark, CC398 is the most common type of LA-MRSA in pigs (Aarestrup et al., 2010). The increasing prevalence of LA-MRSA 398 in Danish pigs and patients has been caused by clonal expansion of the predominant lineages L1, L2, and L3 (Sieber et al., 2018). In the present study, three pork isolates and four chicken isolates of CC398 were located in lineages L1 and L2, while all sushi isolates clustered in the human-associated lineage (Figure 3). Further, livestock-associated CC398 isolates from retail meat and pasta salad did not encode *scn*, whereas isolates from sushi harbored this gene. Even though LA-MRSA CC398 is present in food products, epidemiological data suggest that food plays only a minor role in infections or colonization in humans (Larsen et al., 2015). So far, food as an important transmission pathway for livestock-associated CC398 has only been shown for the CC398/CC9 hybrid of spa-type t899 (Larsen et al., 2016), which was not observed in the present study.

*Staphylococcus aureus* CC5 is a well-described CA-MRSA lineage where MSSA clones could eventually evolve to MRSA through the acquisition of the *mecA* gene (Rasigade et al., 2010; Monecke et al., 2011). Studies have also found that the majority of *S. aureus* isolates from poultry belong to an avian-associated lineage of CC5, which has emerged from a human-to-poultry host jump and is characterized by numerous signatures of adaptation to the avian host including carriage of the avian φAVβ prophage genes (Lowder et al., 2009; Fitzgerald, 2012). In the present study, the φAVβ genes (SAAV_2008, SAAV_2009) were detected in all CC5 isolates (*n* = 13), which is consistent with all these isolates having been obtained from chicken products.

*Staphylococcus aureus* CC1, CC8, CC30, and CC45 have been described as lineages circulating in the hospital and/or community settings (Stefani et al., 2012; Chatterjee and Otto, 2013). CC30 and CC45 have been identified as the most prevalent group lineages associated with human cases of *S. aureus* bacteremia (SAB) (DANMAP, 2017). In this study, raw chicken or pork were the most common sources for CC1, CC8, and CC30, whereas handled ready-to-eat sushi and pasta salad were the most common sources for CC45. This indicates that the presence of CC45 might be due mainly to human handling.

Recently, the community-associated CC88 MRSA was also found to be involved in two hospital outbreaks in Ireland caused by ST78 and ST88, respectively (Earls et al., 2018). Moreover, CC88 (ST78) also appeared for the first time in 2017 as the most frequently prevalent MRSA in human infections in Denmark (DANMAP, 2017). Since sushi is a manually handled food, sushi-CC88 may originate from the hands of restaurant workers (Li et al., 2019).

CC7, CC9, CC15, and CC101 were identified as both human- and livestock-associated lineage groups. Previous studies reported MSSA isolates of CC7, CC9, CC15, and CC101 in Belgian and English communities (Grundmann et al., 2002; Hallin et al., 2007). Livestock-associated *S. aureus* CC9 has emerged as a cause of bloodstream infections in France (Lamamy et al., 2012). Methicillin-sensitive CC15 has been associated with nasal carriage (Sarkar et al., 2016). These CC types were associated with different types of foods in this study. Thus, CC7 originated from pork and sushi, CC9 from pork and turkey, CC15 from chicken and pasta salad, and CC101 from pasta salad. Thus, the CC7, CC9, and CC15 types might be livestock-associated, but, in the case of CC15 and CC101, their presence might also be due to human handling. Finally, CC20 and CC779 were found in sushi and pasta salad, which also indicates that their presence is due to handling.

### Antimicrobial Resistance Marker Genes

*In silico* detection of antimicrobial resistance marker genes based on bacterial genomes is applicable to many areas of surveillance (Zankari et al., 2012). The antimicrobial susceptibility pattern of *S. aureus* isolates from samples of different food products revealed that all 88 isolates were resistant to at least one antimicrobial, including penicillin, tetracycline, and macrolides (Table 2). The presence of antibiotic resistant markers was much more pronounced among CC398 isolates than among other CC types (Figure 2). Specifically, 48.6% (36/74) of MSSA isolates contained the *blaZ* gene encoding penicillin resistance, and 37.8% (28/74) of the MSSA and 78.5% (11/14) of the MRSA isolates harbored tetracycline-resistance genes [*tet*(K) (8), *tet*(L) (1), and *tet*(M) (18) in MSSA; *tet*(K) (8), *tet*(L) (6), and *tet*(M) (9) in MRSA].

**TABLE 2:** Distribution of antimicrobial resistance genes according to sensitivity towards methicillin (MSSA vs. MRSA) and food products.

		****	****	**Beta-lactam genes**			****	****	****	****	**Resistance genes**
	**No.**	**Aminocyclitol genes^a^**	**Aminoglycoside genes^b^**	**Penicillin^c^**	**Methicillin^d^**	**Fluoroquinolone genes^e^**	**Macrolide, Lincosamide, and Streptogramin B genes^f^**	**Tetracycline genes^g^**	**Trimethoprim genes^h^**	**Chloramphenicol genes^i^**	**conferring resistance to three or more classes of agents (%)**
**Methicillin**											
**sensitivity**											
MSSA	74	13	6	36	0	1	27	26	9	1	21 (23.9)
MRSA	14	8	11	14	14	0	13	11	10	0	14 (100.0)
CC8	3	0	3	3	3	0	3	0	0	0	3 (100.0)
CC9	2	0	2	2	2	0	2	2	2	0	2 (100.0)
CC398	9	8	6	9	9	0	8	9	8	0	9 (100.0)
**Food product**											
Chicken	29	4	5	12	4	1	10	13	5	1	11 (12.5)
Pork	17	4	1	10	2	0	5	4	4	0	4 (4.5)
Turkey	15	13	7	15	8	0	15	15	8	0	15 (17.0)
Sushi	17	0	4	8	0	0	10	4	0	0	4 (4.5)
Pasta salad	10	0	0	5	0	0	0	1	2	0	1 (1.1)
Total	88	21	17	50	14	1	40	37	19	1	35 (39.8)

*^*a*^*spc* gene. ^*b*^*ant(6)-Ia-like*, *aph(3′)-III*, *aadD-like*, and *str* genes. ^*c*^*blaZ* gene. ^*d*^*mecA* gene. ^*e*^*qnrB19* gene. ^*f*^*erm(A)*, *erm(B)*, *erm(C)*, *erm(T)*, *vga(E)*, *lnu(A)*, *lnu(B)*, *mph(C)*, and *msr(A)* genes. ^*g*^*tet(K)*, *tet(L)*, and *tet(M)* genes. ^*h*^*dfrA1*, *dfrK*, and *dfrG* genes. ^*i*^*catA1 gene.**

*Staphylococcus aureus* isolated from meat samples in retail outlets is frequently resistant to ampicillin, tetracycline, penicillin, and erythromycin (Kelman et al., 2011; Waters et al., 2011; Wu et al., 2018). Similarly, genes indicating resistance to β-lactam antimicrobials, tetracycline, and erythromycin were observed in the present study. This may be attributed to the fact that tetracyclines, penicillins, and macrolides represent three of the top four classes of antimicrobials used in Danish pig production (DANMAP, 2017).

### Virulence Marker Genes

Toxins constitute an important group of *S. aureus* virulence factors (Yang et al., 2018), with enterotoxins being important to food safety. The distribution of virulence genes among CC types showed the opposite situation to that observed for resistance markers, with CC398 overall encoding much fewer such genes than the remaining CC types (Figure 3). The fact that the majority of ST398-MRSA isolates are negative for major virulence factors such as enterotoxins, Panton-Valentine leucocidin, toxic shock syndrome toxins, and exfoliative toxins has been noted previously (Kadlec et al., 2009; Feßler et al., 2010, 2011; Monecke et al., 2011). In our study, genes encoding enterotoxins were found in 55 isolates, with a prevalence of 67.6% (50/74) in MSSA and 35.7% (5/14) in MRSA, higher than found by Hammad et al. (2012) but lower than reported in other studies (Vázquez-Sánchez et al., 2012; Yang et al., 2018). Some or all of the *seg*, *sei*, *sem*, *sen*, *seo*, and *seu* genes were frequently present in MSSA and also in two MRSA isolates, while the *sea*, *seb*, *sec*, *she*, and *sel* genes were only observed in MSSA isolates. No exfoliative toxin genes (*eta*, *etb*) were found among the 88 isolates (Table 3). Three *lukS/F-*PV-positive CC8 isolates were identified in retail chicken among a total of 88 isolates. Isolates encoding PVL have also been reported in other studies of ready-to-eat food, and a high PVL carriage rate (60.9%) among MRSA isolates has been observed for food in China (Pu et al., 2009; Hanson et al., 2011; Wang et al., 2014; Yang et al., 2018).

**TABLE 3:** Distribution of virulence markers according to sensitivity toward methicillin (MSSA vs. MRSA) and food products.

															**Exfoliative**		**Toxic shock**	**Other virulence**		**SEs**
		**Enterotoxins**													**toxins**		**syndrome**	**markers**		**positive^a^**
						
	**No.**	***sea/sep***	***seb***	***sec***	***seg***	***seh***	***sei***	***sek***	***sel***	***sem***	***sen***	***seo***	***seq***	***seu***	***eta***	***etb***	***tst***	***lukF-PV***	***scn***	**(%)**
**Methicillin**																				
**sensitivity**																				
MSSA	74	15	1	4	33	4	33	1	4	33	29	29	1	32	0	0	1	0	28	50 (67.6)
MRSA	14	0	0	0	2	0	2	3	0	2	2	2	3	2	0	0	2	3	3	5 (35.7)
**Food product**																				
Chicken	29	4	0	0	14	2	14	3	0	14	10	10	3	13	0	0	0	3	4	23 (79.3)
Pork	17	0	0	0	10	1	10	0	0	10	10	10	0	10	0	0	0	0	2	11 (64.7)
Turkey	15	5	0	0	2	0	2	0	0	2	2	2	0	2	0	0	2	0	0	7 (46.7)
Sushi	17	5	1	2	6	1	6	1	2	6	6	6	1	6	0	0	1	0	16	11 (64.7)
Pasta salad	10	1	0	2	3	0	3	0	2	3	3	3	0	3	0	0	0	0	9	3 (30.0)
Total	88	15	1	4	35	4	35	4	4	35	31	31	4	34	0	0	3	3	31	55 (62.5)

*^*a*^Numbers in parentheses indicate the prevalence of enterotoxin genes in each category.*

A total of 31 isolates were confirmed to contain *scn* genes, including 25 isolates from sushi and pasta salad products. Thus, the *scn* gene was observed in fifteen sushi isolates with the types CC1 (1), CC7 (4), CC20 (2), CC45 (4), CC398 (4), and ST2867 (1), nine pasta salad isolates with the types CC15 (2), CC30 (1), CC45 (2), CC101 (3), and CC779 (1), four chicken-isolates with the types CC8 (3) and CC15 (1), and two pork-isolates with the types CC 1(1) and CC45 (1). The presence of *scn* genes in at least sushi and pasta salad isolates indicates that they likely originated from a human reservoir as food contaminants.

The *tst* gene encoding toxic shock syndrome toxin 1 (*TSST-1*) was detected in two CC398 isolates from turkey meat and one CC45 isolate from a sushi product. Raw reads were mapped to the *tst* reference gene (GenBank accession no. AP009324.1) to confirm its presence in the CC398 isolates, and assemblies of the gene regions were aligned to the reference sequence of the SaPI1 carrying the *tst* gene (Lindsay et al., 1998). This showed that the two CC398 isolates carried almost identical elements, indicating a single acquisition, while they both only shared some similarity with the reference sequence, indicating that the *tst* genes were carried on a SaPI-like element. The two isolates clustered together in the phylogenetic tree, which also makes a single acquisition of the mobile genetic element carrying the genes encoding *TSST-1* likely. The source of the two isolates was turkey meat sold in a Danish supermarket with limited information about the country of origin (Tang et al., 2017). The toxic shock syndrome toxin is a super-antigen that can cause a variety of symptoms (Schlievert, 1993; Kare and Dang, 2008). This study shows for the first time to our knowledge the presence of *tst* in *S. aureus* CC398.

## Conclusion

Our study takes a combined bioinformatical approach to acquire detailed data on antimicrobial-resistant and virulence markers in *S. aureus* isolated from retail meat and ready-to-eat foods in Copenhagen, Denmark. Thirteen lineages were found to be present in food products, with the most commonly detected isolates belonging to CC398 and CC5. CC398 was identified as the most commonly encountered clonal complex (*n* = 31). CC5 isolates (*n* = 13) were identified as an avian-associated lineage containing avian φAVβ prophage genes. MRSA isolates were detected among CC398 (*n* = 9), CC9 (*n* = 2), and CC8 (*n* = 3). Resistance genes towards β-lactam antimicrobials, tetracycline, and erythromycin were frequently observed, which correlates with these three antimicrobial classes being commonly used in Danish livestock production. The *tst* virulence gene was detected in one CC45 and two CC398 isolates. The presence of the *tst* gene encoding the *TSST-1* toxin in CC398, reported here for the first time, was most likely the result of a single acquisition of a SaPI-like mobile genetic element. The sushi-CC398 isolates carrying the *scn* gene likely originated from a human reservoir (human-associated lineage), while the other isolates originated from livestock. Together, our results show that both human and animal reservoirs can contribute to contamination in food products and that retail foods may serve as a vehicle of *S. aureus* between livestock and humans.

## Data Availability Statement

The whole-genome sequence data generated in this study have been submitted to the European Nucleotide Archive under BioProject accession number PRJEB32298.

## Author Contributions

HL, JL, HI, and AD designed the study. HL and NS performed the experiments. HL, PA, MS, and RS analyzed the bioinformatics data. HL and JL wrote the manuscript.

## Conflict of Interest

The authors declare that the research was conducted in the absence of any commercial or financial relationships that could be construed as a potential conflict of interest.

## Footnotes

1 http://www.mgc.ac.cn/VFs/

2 https://en.ssi.dk/

3 www.danmap.dk

4 www.mlst.net

5 https://cge.cbs.dtu.dk/services/CSIPhylogeny/

6 https://itol.embl.de/

7 http://genomicepidemiology.org/
